# Azithromycin resistant gonococci: a literature review

**DOI:** 10.1186/s13756-020-00805-7

**Published:** 2020-08-18

**Authors:** Awoke Derbie, Daniel Mekonnen, Yimtubezinash Woldeamanuel, Tamrat Abebe

**Affiliations:** 1 Department of Medical Microbiology, College of Medicine and Health Sciences, Bahir Dar University, Bahir Dar, Ethiopia; 2 Centre for Innovative Drug Development and Therapeutic Trials for Africa (CDT-Africa), Addis Ababa University, Bahir Dar, Ethiopia; 3 Department of Health Biotechnology, Biotechnology Research Institute, Bahir Dar University, Bahir Dar, Ethiopia; 4 Department of Medical Microbiology, Immunology and Parasitology, School of Medicine, College of Health Sciences, Addis Ababa University, Addis Ababa, Ethiopia

**Keywords:** *Neisseria gonorrhoeae*, Gonococcus, Azithromycin, Resistance

## Abstract

**Objective:**

Gonorrhea is the second most common sexually transmitted bacterial infection (STI) next to Chlamydia. Untreated cases could results in major complications like pelvic inflammatory disease (PID), ectopic pregnancy, infertility, miscarriage, fetal death and congenital infections. Gonorrhea has been treated with antibiotics for more than eight decades. However, the emergence and spread of antimicrobial resistance (AMR) in gonococcus seriously compromises the management of the disease. The aim of this review was to describe the current developments in the field of azithromycin resistant gonococci.

**Methods:**

Literatures published in English in the last 10 years were retrieved from PubMed, SCOPUS, Google scholar, Cochrane library and the Google databases using relevant searching terms.

**Results:**

Gonococcus is capable of using a number of strategies to confer resistance as the bacterium has an extraordinary capacity to alter its genome. So far the accumulated data on the field showed that the world is heading towards a pandemic of extensively drug-resistant (XDR) gonococcus which is now seems to be evolving into a true *“superbug”.* Hence, in the near future gonorrhea may become untreatable on the international basis unless new drugs become available.

An antibiotic resistance in gonococcus has been noted beginning in 1940s against sulfonamides. Since then, resistance has rapidly emerged to penicillins, tetracyclines, macrolides, fluoroquinolones, and cephalosporins. Currently, in most nations, the injectable extended-spectrum cephalosporin (ESC), i.e. ceftriaxone based therapy is the only remaining option for gonorrhea. Based on the WHO and the US-CDC recommendations, countries are increasingly using a combination of cephalosporin and azithromycin for the treatment of gonorrhoea. Azithromycin revolutionized gonoccocal therapy as it shortened treatment time by more than half from 7 to 14 days and improved patient compliance due to high tissue levels and long half-life. However, constantly emerging reports from different parts of the globe showed that *N. gonorrhoeae* is developing significant level of resistance against azithromycin, and so far more than 33% level of resistance was reported. Two strategies have been commonly implicated in gonococcal resistance against azithromycin: over expression of an efflux pump (due to mutations at *mtrR* coding region) and decreased antimicrobial affinity (due to mutations in genes encoding the 23S ribosomal subunit).

**Conclusions:**

With no alternative antimicrobial treatment options for gonorrhoea and only a few new drugs in the development pipeline, it is necessary to monitor drug resistance and optimize treatment regimens regularly. Moreover, investigations for novel drugs should be wired.

## Introduction

Annually an estimated 78 million new cases of gonorrhea occurred among people aged 15–49 years around the world. About 75–85% of the cases were in developing countries [1]. A sharp decline of gonorrhea incidence has been noted in many developed countries, but the disease remains one of the most common STIs in the developing world [1, 2]. *Neisseria gonorrhoeae* (Gonococcus)*,* which is the etiological agent of gonorrhea, typically colonizes and infects the genital tract in men and women, but may be found in additional body sites such as the rectal and oropharyngeal mucosa with or without clinically evident infection [3–5]. It is transmitted through sexual contact and can also be passed from mother to child through infected birth canal [6–9].

Gonorrhea can be prevented by exercising safe sexual practice like, using condom and the disease is treatable and medications have been used for more than eight decades [10]. However, treatment has been greatly compromised by the emergence of drug resistance by *N. gonorrhoeae* for every drug used to treat it [1, 10]. Because of its high affinity for horizontal gene transfer, antibiotic resistant gonorrhea is seen as an emerging public health threat around the globe [3, 11]. The irrational and unwise use of antibacterial agents increases the development of antibiotic resistance in gonorrhoea as well as other bacterial diseases [5], especially in the developing countries [1, 7, 12].

Since the introduction of antimicrobial treatment, resistance has rapidly emerged to sulphonamides, penicillins, tetracyclines, macrolides (like, azithromycin), fluoroquinolones, and early-generation cephalosporins. Currently, in most nations, the injectable extended-spectrum cephalosporin (ESC) ceftriaxone based therapy is the only remaining empiric treatment for gonorrhea. However, gonococcal in-vitro resistance and/or treatment failures to the last-line oral ESC cefixime and, more rarely, to ceftriaxone have been verified in many countries. Consequently, dual antimicrobial therapy, mainly ceftriaxone plus azithromycin is under practice in most countries [8, 10, 13–15]. Resistance of *N. gonorrhoeae* to azithromycin slowly developed since 1990 in some areas of the world and then a number of similar reports came to the front globally [14, 16–21].

Azithromycin was developed as a synthetic derivative of erythromycin in 1980s. It binds to the 50S ribosome subunit preventing translocation of the peptidyl-tRNA, causing ribosomes to release incomplete polypeptides. This results in a bacteriostatic effect [22]. It had a significantly higher activity than erythromycin against *N. gonorrhoeae* [23]. However, studies in different parts of the globe showed that the world community is facing challenge due to significant level of drug resistance to azithromycin by some strains of gonococci [14, 23–28] and reports are showing that azithromycin resistance tended to increase [29, 30].

Standardized monitoring of the antimicrobial susceptibility profile has been restricted to some nations in the developed world; such as the Gonococcal Isolate Surveillance Project (GISP) in the US and Gonococcal Resistance to Antimicrobials Surveillance Program (GRASP) in England and Wales [7] although some fragmented reports are available in Africa [31, 32]. A recent review by Tilahun et al*,* (2017) showed that antimicrobial resistance (AMR) data is not at all available for > 40% of the African countries. Plus, the level of resistance to commonly prescribed antibiotics, including azithromycin*,* is significantly higher [33]. Surveillance of the antimicrobial resistance, especially for the drugs of first choice: i.e. ceftriaxone and azithromycin, becomes very important in monitoring the emergence and spread of resistance [14, 32, 34]. With the above background, the aim of this review was to describe the current developments on azithromycin resistant gonococci. It is also imperative that changes in resistance patterns of gonococci be monitored continually to ensure optimal treatment both of the individual patients and for maintaining the efficacy of empiric therapy regimes. With the above background, the aim of this review was to describe the current developments on azithromycin resistant gonococci.

## Methods

In this review we considered peer-reviewed journal articles, governmental documents and unpublished articles (thesis) reported in English language since 2010. Observational quantitative studies that reported the level of azithromycin resistant gonococci among individuals presumptive or confirmed for gonorrhea irrespective of gender and the age group were consulted. PubMed/Medline, SCOPUS, Google scholar, Cochrane library and the Google database were source of the literatures. Combinations of the following key terms were considered to retrieve the articles; <*N. gonorrhea/*gonococcus, azithromycin resistant *N. gonorrhea/*gonococcus*,* antimicrobial resistance in gonococcus, epidemiology of azithromycin resistant gonococcus>. Studies reported before 2010 were excluded.

## Results

### Action of azithromycin

Azithromycin is a macrolide antibiotic and the sole member of the azalide subclass. It is derived from erythromycin; it has an aza-methyl substitution (insertion of a nitrogen atom) in the macrolide ring. This addition increases the stability with no change on the mechanism of action. The addition of the second amine group resulted in important advantages over erythromycin, including greater tissue penetration and an extended half-life. Azithromycin binds to the 50S ribosome subunit preventing translocation of the peptidyl-tRNA, blocking the peptide exit channel in 50S subunits by interacting with 23S rRNA, and causing ribosomes to release *incomplete polypeptides*. This results in a bacteriostatic effect. Azithromycin demonstrate in vitro against a wide range of bacteria including gonococcus [22].

### Azithromycin resistance

Resistance of *N. gonorrhoeae* to azithromycin slowly developed since 1990 in some areas of the world and then a number of similar reports came to the front globally [14, 16–21] and the reported prevalence varies across the nations (Table 1). Some countries like the US started Azithromycin susceptibility surveillance testing in 1992 and continue today as part of their AMR monitoring for early detection and action [8]. Likewise, the European Gonococcal Antimicrobial Surveillance Programme (EURO-GASP) was also established by in 2004 in response to the emerging antimicrobial resistance of *N. gonorrhoeae* [29]. In contrast, in most African countries there is no strong AMR surveillance not only for Azithromycin, but also for other antimicrobials to evaluate the temporal and spatial distribution and epidemiology of AMR in the continent [33].

**Table 1:** Magnitude of Azithromycin resistant gonococci measured by different methods

Country	Author	Year of the study	Method of AST	Magnitude of AZM resistance (%)	Reference
Hungary	Brunner et al. Brunner et al.	2014, 2016	MIC strip test Liofilchem®, Agar dilution	16–30.0	[14, 26]
India	Kulkarni et al.	2018	Disc diffusion with E-test	5	[24]
China	Jing-Yao et al.; Li, W et al. Jiang et al. Yin et al.	2016, 2017, 2017 2018	Agar dilution method	3.6–28.6	[35–38]
Europe (EU)	Cole et al.	2014	Agar dilution	5.3	[39]
United States	Kirkcaldy et al,	2015	Agar dilution methods	0.4	[40]
Germany	Regnath et al. Beder et al.	2016 2018	E-test	5.5–11.9	[18, 29]
Netherlands	Wind et al.	2017	Agar dilution	1.2	[25]
Zimbabwe	Latif et al.	2016	E-test	10	[31]
Uganda	Vandepitte et al.	2014	E-test	16.2	[32]
Poland	Młynarczyk-Bonikowska et al.	2014	E-test	Sensitivity was at 38.5%	[41]
South America and the Caribbean	Dillon J-AR et al.	2014	Agar dilution	10	[42]
France	Belkacem et al.	2016	E-test	1	[16]
Cyprus	Cole et al.	2015	Agar dilution	33.3	[17]
Greece		2015	Agar dilution	22.7	
Taiwan	Liu et al.	2018	Agar dilutiom	14.6	[20]
Australia	Lahra et al.	2016	Agar dilution	1.7	[21]

Resistance to azithromycin was defined as an MIC > 0.5 mg/L [43]

Gonococci are known to use multiple mechanisms of antibiotic resistance. Genetically these changes may be mediated by either chromosomal or extra-chromosomal elements (plasmids) [7]. It is assumed that many of the resistance mechanisms evolve from specific mutations from commensal Neisseria species that live in the oral cavity. Because *N. gonorrhoeae* is uniquely capable of assimilating external DNA during its life cycle, the transfer of chromosomally encoded resistance genes from commensal Neisseria species that have acquired resistance to various antimicrobials is rapid and extensive [8].

Many cases of macrolide resistance in clinical strains were linked to alteration of specific nucleotides in 23S rRNA within the large ribosomal subunit. Generally, bacterial resistance to macrolides may result from modification of the ribosomal target by either rRNA methylase-associated modification of the 23S rRNA or specific mutations in the 23S rRNA and/or from an over expressed efflux pump system. The rRNA methylases can cause macrolide resistance through blocking of macrolide binding to 23S rRNA by methylating an adenosine residue at position 2058 (*E. coli* numbering system), which is located in peptidyl transferase domain V. Genes encoding rRNA methylase (referred to as macrolide-lincosamide-streptogramin B resistance genes, or *erm* genes) can be carried by conjugative transposons [10, 22, 44–46]. It is suggested that high level azithromycin resistant isolates were descendants of the low-level azithromycin resistant isolates and, accordingly, azithromycin exposure might provide the selection pressure for emergence of the high level azithromycin resistant phenotype [43, 47].

A recent study in UK by Chisholm and his research team have demonstrated that high-level azithromycin resistance in gonococci result from a single point mutation (A2059G) in the peptidyltransferase loop in domain V of the 23S rRNA gene. Mutation of a single allele is insufficient to confer high-level azithromycin resistance, but it can develop under selection pressure [48]. Similarly according to Wind et al. study (2017), exposure to azithromycin was significantly associated with A39T or G45D *mtrR* mutations. This implies that, frequent azithromycin use in populations at high risk of contracting *N. gonorrhoeae* induces an increase in minimum inhibitory concentration (MIC), and may result in resistance [40, 49, 50]. A similar study in France also showed that mutations in domain V of 23S rRNA, encoded by the *rrl* gene, were associated with *Azithromycin* resistance by gonococcus [16].

Shigemura and his research team in Japan likewise reported that a deletion mutation of the *mtrR* promoter region is a possible mechanism of azithromycin resistance and may be a significant indicator for higher MICs (0.5 μg/ml or higher) in *N. gonorrhoeae* infection [51]. A recently released data showed a decreased antimicrobial susceptibility of gonococci to azithromycin that may limit effectiveness of the drug [52, 53]. The reduced azithromycin susceptibility has arisen through multiple mechanisms [54] as described above. Resistance to all major groups of antibiotics has arisen hand in hand with their extensive use in medicine and animal husbandry.

The two common antimicrobial susceptibility testing methods for gonococci are phenotypic and molecular techniques (based on the detection of specific antimicrobial resistance gene and mutations associated with resistance phenotypes). Disk diffusion and test for minimal inhibitory concentration (in the form of agar dilution, broth microdilution, agar gradient dilution (E-test)) methods are the two major phenotypic methods that commonly done in most laboratories around the world as these methods are relatively cheap and simple [55, 56] (Table 1).

### Future perspectives

The only effective option for treating gonorrhea and stopping its spread has been the use of antimicrobial therapy. Currently, there is no vaccine to prevent gonococcal infection. Antimicrobial treatment options have diminished over time due to the progressive emergence of AMR to drugs previously used to treat gonorrhea. As a result, now we are left with limited options for treating gonorrhea. At this time, a number of studies are supporting the continued use of azithromycin in a combination therapy regimen for gonorrhea. However, the use of azithromycin in dual therapy for gonorrhoea has been increasingly questioned during the past few years, because of increasing azithromycin resistance in many countries [40, 43, 49]. Hence, we might not be able to rely on azithromycin to protect ceftriaxone [43]. Moreover, the emergence of extremely drug resistant (XDR) gonorrhoea argues for enhanced efforts to develop novel antimicrobial agents and a gonococcal vaccine [57]. It is also advised to exercise rational use of drugs and to promote continued antimicrobial stewardship for gonorrhea [58].

Studies are under way to look for new treatment modalities for the treatment of gonorrhea due to isolates possessing resistance to ceftriaxone and azithromycin; one of such studies is the use of fosfomycin alone and in combination with ceftriaxone or azithromycin [59]. In another study by Pettus and his colleagues, an in vitro assessment of azithromycin in combination with gentamicin demonstrated inhibition of growth and suggests that clinical trials may be warranted to assess the utility of this combination in treating gonorrhea infections [60]. Su et al. in China also tested the activity of a DNA Gyrase Inhibitor, ETX0914 against *N. gonorrhoeae* and suggested that it may be an effective treatment option for gonorrhea [52]. In a phase II trial of oral Solithromycin for the treatment of gonorrhea (2015), authors reported that single dose solithromycin, in doses of 1000 and 1200 mg was 100% effective for treatment of culture-proven gonorrhea at genital, oral and rectal sites of infection and is a promising new agent for gonorrhea treatment [61].

The capacity and ease for genetic recombination and high transmissibility of resistant genes has made the development of new antibiotics a challenging area of research [39]. The choice of antibacterial agent must take into account the data generated by laboratory based surveillance of susceptibility. This surveillance would be useful not only in deciding the correct treatment but also in helping to detect the emergence of new antibiotic resistant traits and to monitor the effectiveness of prescribed treatment [7].

For better understand the molecular basis of *N. gonorrhoeae* infection, extensive genome sequencing studies have to be conducted on a diverse collection of strains from different geographical locations and collected over longer time periods [62]. This knowledge will facilitate disease prevention, surveillance and control, improve diagnostics and may help to facilitate the development of effective vaccines or new therapeutics.

## Conclusions

Currently most countries around the globe are using dual therapy for the treatment of gonorrhea; ceftriaxone and azithromycine. The rationale for gonococcal combination therapy includes using different antimicrobials with different mechanisms of action to potentially mitigate the spread of antimicrobial resistance. There are a number of studies conducted on the AMR profile of gonococci and reported greater than 33% level of azithromycin resistance globally. Gonococcus becomes resistant for this drug using different mechanisms. With no alternative antimicrobial treatment options for gonorrhoea and only a few new drugs in the development pipeline, AMR to extended spectrum cephalosporins and Azithromycin among *N. gonorrhoeae* is a major public health concern. So, ongoing surveillance and public health action are necessary to monitor trends in antimicrobial resistance, and rapidly assess clinical treatment failures. New antimicrobials are currently being assessed and further research should investigate alternative antimicrobial combinations, which will be essential to provide optimum therapy against this formidable pathogen.

Moreover, it has become substantive to initiate and sustained national and international efforts to reduce misuse of antibiotics so as to prevent further emergence and spread of antimicrobial resistance. It is necessary not only to monitor drug resistance and optimize treatment regimens, but also to gain deeper insight into how gonococcus develops drug resistance. Studies could also help in finding out new drug targets in *N. gonorrhoeae* and also a possibility of identification of new drugs for treating gonorrhoea.

## Data Availability

All the generated data in this review are included in the manuscript.

## Abbreviations

AMR: Antimicrobial Resistance
ESC: Extended-spectrum Cephalosporin
MIC: Minimum inhibitory concentration
PID: Pelvic Inflammatory Disease
STD: Sexually Transmitted Disease
STI: Sexually Transmited Infection
tRNA: Transfer Ribonucleic acid
WHO: World Health Organization
